# “Cherry blossom” in Kyoto brought hope to patients with chronic hepatitis B infection

**DOI:** 10.1007/s12072-024-10705-2

**Published:** 2024-08-27

**Authors:** Jing Chen, Ji-Dong Jia, Hui Zhuang, Yu-Mei Wen, Shiv Kumar Sarin, Masao Omata, George Lau, On behalf of APASL Viral Elimination Taskforce (in no particular order), George Lau, Masao Omata, Jidong Jia, Hui Zhuang, Yu-Mei Wen, Xinxin Zhang, Jin Mo Yang, Tawesak Tanwandee, Diana Payawal, Saeed Hamid, Shiv Kumar Sarin, Rakhi Maiwall, Manoj Kumar, Jing Chen, Dong Ji, Wenhong Zhang, Fusheng Wang, Jiangao Fan, Lungen Lu, Xiaoguang Dou, Xiaolong Qi, Qin Ning, Ming-Lung Yu, Jacob George, George B. B. Goh, Sang Hoon Ahn, Rino Alvani Gani, Mohd Ismail Merican, Khin Maung Win, Oidov Baatarkhuu, Hasmik Ghazinyan, Manal H. El-Sayed, Anuchit Chutaputti, Phunchai Charatcharoenwitthaya, Pei-Jer Chen, Jia-Horng Kao, Rosmawati Mohamed, Rakesh Aggarwal, Alexander Thompson, Hong You, Hong Ren, Jian Sun, Yoon Jun Kim, Grace Wong, Fu Gao, Gang Li, Jun-Qi Niu, Yu Wang, Zhi-Liang Gao

**Affiliations:** 1 JC School of Public Health and Primary Care, Faculty of Medicine, Chinese University of Hong Kong, Hong Kong SAR, China; 2 Liver Research Center, Beijing Friendship Hospital, Capital Medical University, Beijing, China; 3 Department of Microbiology and Centre for Infectious Diseases, Peking University Health Science Centre, Beijing, China; 4 Key Laboratory Medical Molecular Virology, Ministry of Education/Health, School of Basic Medical Sciences, Shanghai Medical College, Fudan University, Shanghai, China; 5 Department of Hepatology, Institute of Liver and Biliary Sciences, New Delhi, India; 6 Yamanashi Hospitals (Central and Kita) Organization, Kofu-Shi, Yamanashi, Japan; 7 Humanity and Health Clinical Trial Center, Humanity and Health Medical Group, 14F/21F, 9 Queen’s Road Central, Central, Hong Kong SAR, China; 8 Liver Cancer Institute, Zhongshan Hospital, Fudan University, Shanghai, China

**Keywords:** WHO guidelines, Chronic Hepatitis B, HBV treatment expansion, Liver cancer prevention

At the recent 33rd annual meeting of Asia–Pacific Association for the Study of Liver held at Kyoto, Japan (27th–31st March, 2024), a momentous step with far-reaching impact has been initiated by the launching of the new World Health Organization (WHO) guidelines on the management of chronic hepatitis B (CHB) infection [1, 2]. The new WHO guidelines, advocate for bold recommendations to treat all unless normal ALT and HBV DNA < 2000 IU/ml (if assay available) and no liver fibrosis and other risk factors. For over two decades, despite the availability of effective and low-cost measures in screening, treatment and cancer surveillance, there has been a considerable gap in bringing these services to our CHB patients. Consequently, little if any significant decline in the age-standardized incidence and mortality related to liver cancer has occurred. The annualized rate of change of global HBV-related mortality rate has remained almost unchanged at less than 0.4% between 2015 and 2017 [3]. One of the key issues contributing to this stagnation is the complexity of major HBV guidelines [4], which have imposed undue hurdles for policymakers in recognizing the gravity of “under-diagnosis”, “under-treatment” and “under-surveillance of cancer” in CHB patients, leading to a devasting escalation of hepatocellular carcinoma-related deaths worldwide. These guidelines were formulated based on limited research evidence rather than real-world big data, requiring robust and precise laboratory techniques which are not widely accessible to the resource-limited regions [4]. According to a recent cost-effectiveness analysis, based on decision-tree Markov state-transition model, expanding antiviral treatment to HBV-infected patients with ALT thresholds of 30 U/L for males and 19 U/L for females, with 80% treatment coverage for HBsAg-positive individuals aged 18–80 years, will be required to significantly diminish HBV-related morbidity and mortality [5]. This strategy, coupled with lowered generic drug costs and revised medical insurance subsidization policies, particularly in HBV-endemic countries like China, has the potential to significantly reduce HBV-related complications and deaths, aligning the global target of 65% reduction in HBV-related death [6]. These new WHO guidelines with the expanding treatment criteria were echoed by the HBV guidelines published by China [7] and APASL Viral Elimination Taskforce [8]. Hopefully, in the very near future, as nations embrace these guidelines and scale-up diagnosis and treatment to achieve the WHO hepatitis elimination targets, we will witness a curtail of suffering of our chronic hepatitis B patients and their love ones, offering hope akin to the blossoming cherry trees in Kyoto!
